# Radiographic and Clinical Adjacent Segment Disease After Posterior Instrumented Fusion for Traumatic Thoracolumbar and Lumbar Vertebral Fractures

**DOI:** 10.3390/jcm15145416

**Published:** 2026-07-10

**Authors:** Ali Bülent Baz, Vugar Nabi, Fatih Duygun, Ömer Faruk Kılıçaslan, Aliekber Yapar, Mehmet Ali Tokgöz

**Affiliations:** 1 Department of Orthopaedics and Traumatology, Antalya Training and Research Hospital, University of Health Science, 07100 Antalya, Türkiye; vugar.nabi@sbu.edu.tr (V.N.); fduygun2000@yahoo.com (F.D.); kilicaslanfaruk@hotmail.com (Ö.F.K.); 2 Department of Orthopaedics and Traumatology, Faculty of Medicine, Akdeniz University, 07100 Antalya, Türkiye; aliekberyapar@hotmail.com; 3 Department of Orthopaedics and Traumatology, Faculty of Medicine, Gazi University, 07100 Ankara, Türkiye; m.alitokgoz@gmail.com

**Keywords:** adjacent segment disease, spinal fusion, thoracolumbar spine, spinal fractures, oswestry disability index, quality of life

## Abstract

**Background:** Adjacent segment disease (ASD) after spinal fusion is well described in degenerative cohorts, but its radiographic and clinical behavior after acute traumatic thoracolumbar fracture fusion remains incompletely defined. This study evaluated whether ASD after traumatic fusion represents an imaging finding alone or a clinically relevant recovery-limiting event. **Methods:** This retrospective study included 98 patients who underwent posterior instrumented thoracolumbar/lumbar fusion for acute traumatic vertebral fractures. Because urgent surgery precluded meaningful preoperative disability assessment, the 1-month postoperative Oswestry Disability Index (ODI) was used as an early post-treatment functional reference point. Radiographic ASD was defined by new or progressive adjacent-level disc degeneration, disc-space narrowing, translation, angular instability, or junctional kyphosis, whereas clinical ASD was defined strictly as revision-confirmed symptomatic adjacent pathology. **Results:** Radiographic ASD occurred in 54 patients (55.1%), whereas revision-confirmed clinical ASD occurred in 11 patients (11.2%). Proximal and distal involvement were observed in 39.8% and 36.7% of patients, respectively. ODI improved from 42.5 ± 15.4 at 1 month to 27.0 ± 10.9 at final follow-up. Radiographic ASD alone was not associated with worse functional recovery. In contrast, revision-confirmed clinical ASD identified patients with markedly reduced ODI improvement (4.4 ± 2.8 vs. 16.9 ± 17.0; *p* = 0.007), and no patient with revision-confirmed clinical ASD achieved the ODI minimum clinically important difference. **Conclusions:** ASD after traumatic fusion should be interpreted as a clinically stratified phenomenon: frequently radiographic, but clinically relevant primarily when revision-confirmed and associated with reduced ODI-based recovery.

## 1. Introduction

Adjacent segment degeneration and adjacent segment disease represent two related but clinically distinct consequences of spinal fusion. Adjacent segment degeneration refers to radiographic deterioration at the motion segments immediately above or below a fused construct, including disc space narrowing, facet arthrosis, instability, listhesis, stenosis, or adjacent vertebral collapse. Adjacent segment disease, in contrast, implies that these structural changes have become clinically meaningful, producing new pain, neurological symptoms, functional deterioration, or the need for revision surgery [1,2,3]. Although the distinction appears semantic, it is central to outcome interpretation: radiographic degeneration may remain clinically silent, whereas symptomatic ASD may substantially compromise health-related quality of life, patient satisfaction, and the perceived success of an otherwise technically successful fusion.

A further challenge in ASD research is the imperfect relationship between imaging findings and symptoms. Radiographic spinal degeneration is common, increases with age, and may be observed even in asymptomatic individuals; therefore, imaging abnormalities should be interpreted in the context of pain, neurological symptoms, functional limitation, and need for treatment rather than as disease-defining findings in isolation [4]. This distinction is particularly important in adjacent segment pathology, where radiographic adjacent segment degeneration, symptomatic adjacent segment disease, and reoperation-confirmed clinical failure are related but non-identical endpoints [5].

The biological and biomechanical basis of ASD has been extensively investigated in degenerative lumbar fusion cohorts. Instrumented fusion eliminates motion at the treated level and redistributes mechanical load to the remaining mobile segments, thereby increasing intradiscal pressure, facet loading, shear stress, and compensatory hypermobility at adjacent levels [1,2,3,6,7]. Reported risk factors include older age, high body mass index, pre-existing disc or facet degeneration, osteoporosis, fusion length, sagittal malalignment, floating fusion, laminectomy or facet violation adjacent to the construct, excessive disc space distraction, and nonunion [8,9,10,11,12,13,14,15]. Long-term studies have further suggested that ASD is not a uniform entity but rather a spectrum in which patient biology, baseline degeneration, construct stiffness, and postoperative alignment interact over time [16,17]. However, most available evidence has been derived from degenerative conditions such as spondylolisthesis, stenosis, and adult deformity, leaving the post-traumatic fusion population comparatively underexplored.

Traumatic thoracolumbar and lumbar vertebral fractures create a different biological and mechanical environment from elective degenerative fusion. In acute trauma, the spine is subjected not only to surgical immobilization but also to the initial injury itself: endplate disruption, occult disc trauma, posterior ligamentous complex injury, facet capsule damage, paraspinal muscle injury, local kyphotic collapse, and abrupt alteration of regional sagittal mechanics may all “prime” adjacent segments before the first pedicle screw is inserted [17,18]. Moreover, urgent stabilization is frequently performed to restore stability, prevent deformity progression, protect neurological structures, and allow early mobilization. This acute setting limits the opportunity for preoperative functional optimization and may require constructs that cross transitional thoracolumbar zones, sacrifice motion segments, or prioritize fracture reduction over long-term adjacent segment preservation. Therefore, ASD after fracture fusion may not simply represent the accelerated version of degenerative ASD; it may reflect a trauma-specific failure pathway in which the adjacent disc–facet complex has already been biomechanically injured and is then exposed to the increased rigidity of posterior instrumentation.

Recent evidence supports the need to evaluate post-traumatic fusion beyond radiographic union alone. Contemporary studies of thoracolumbar fracture surgery have shown that pain and health-related quality of life improve after fixation or fusion, but functional recovery may extend beyond the early postoperative period and may plateau after several months. Implant retention, prolonged immobilization, and loss of segmental range of motion have also been linked to inferior disability and quality-of-life profiles in selected cohorts. More recently, traumatic lumbar fracture fixation has been associated with measurable adjacent segment degeneration, with some studies suggesting a possible mismatch between radiographic ASD and short-term patient-reported outcomes. This observation raises an important clinical question: is ASD after traumatic fusion truly silent, or does it become clinically visible only when follow-up is long enough and outcomes are assessed with sensitive HRQoL measures?

Accordingly, the present study investigates the incidence and clinical relevance of ASD following posterior instrumented thoracolumbar/lumbar fusion for acute traumatic vertebral fractures. By focusing on a homogeneous surgical trauma cohort and integrating radiographic adjacent-segment changes with ODI-based functional recovery, this study aims to clarify whether ASD represents an incidental imaging finding or a clinically meaningful determinant of long-term recovery. We hypothesized that ASD after traumatic vertebral fracture fusion may not be merely an incidental degenerative imaging finding, but rather a trauma-associated adjacent-segment vulnerability pattern that, when clinically expressed, is associated with residual disability and impaired functional recovery.

## 2. Materials and Methods

### 2.1. Study Design and Patient Selection

This retrospective cohort study included 98 consecutive patients who underwent posterior instrumented thoracolumbar/lumbar fusion for acute traumatic vertebral fractures at a single tertiary spine centre. The study was designed and reported in accordance with the principles of the Strengthening the Reporting of Observational Studies in Epidemiology (STROBE) statement. The primary objective was to determine the incidence of adjacent segment disease (ASD) after posterior instrumented fusion for traumatic vertebral fractures and to investigate its relationship with postoperative disability and health-related quality-of-life-related functional recovery.

Patients were eligible if they had an acute traumatic thoracolumbar or lumbar vertebral fracture requiring surgical stabilization, underwent posterior pedicle screw instrumentation with or without decompression, and had available postoperative clinical and radiographic follow-up data. Surgical indication was based on fracture morphology, mechanical instability, neurological status, posterior ligamentous complex integrity, and the Thoracolumbar Injury Classification and Severity Score (TLICS), with operative treatment generally indicated for unstable injuries and TLICSs greater than 4 [19]. Patients with pathological fractures due to tumour or infection, primary osteoporotic compression fractures, previous fusion involving the index or adjacent levels, incomplete radiographic records, or unavailable postoperative Oswestry Disability Index (ODI) data were excluded.

Because this was an acute trauma cohort and most patients underwent urgent surgery shortly after admission, conventional preoperative ODI assessment was not considered methodologically reliable. In this setting, a preoperative ODI would primarily reflect acute fracture pain, immobilization, emergency admission conditions, and trauma-related distress rather than true pre-injury functional status. Therefore, the 1-month postoperative ODI was defined a priori as the early post-treatment functional reference point.

### 2.2. Ethical Approval

The study was conducted in accordance with the Declaration of Helsinki and was approved by the local institutional ethics committee (IRB-2020-212, 3 July 2020). Written informed consent for the use of clinical and radiographic data was obtained from all participating patients or their legal representatives, according to institutional regulations.

### 2.3. Data Collection

Clinical, demographic, surgical, radiographic, and patient-reported outcome data were extracted from electronic medical records, operative reports, outpatient follow-up records, and institutional radiographic archives. The collected variables included age, sex, body mass index (BMI), smoking status, diabetes mellitus, hypertension, trauma mechanism, fracture level, time from admission to surgery, length of hospitalization, surgical approach, construct extent, number of instrumented vertebrae, use of interbody fusion, operative time, estimated blood loss, perioperative complications, revision surgery, and final follow-up duration.

Trauma mechanisms were categorized as high-energy falls, traffic accidents, simple falls, and other mechanisms. Fracture level was recorded according to the injured vertebra or vertebrae. The construct extent was determined from operative reports and confirmed on postoperative radiographs. Proximal facet violation, interbody fusion, proximal junctional kyphosis (PJK), distal junctional kyphosis (DJK), and radiographic signs of adjacent segment involvement were recorded as separate variables.

### 2.4. Surgical Technique

All patients underwent posterior pedicle screw instrumentation under fluoroscopic guidance. The standard construct included fixation of the vertebrae immediately above and below the fractured segment, with extension of the construct when required by fracture morphology, instability pattern, neurological compromise, bone quality, or surgeon judgement. Pedicle screws were inserted into the fractured and adjacent vertebrae when anatomically and mechanically feasible. Particular attention was paid to preserving the cranial and caudal adjacent facet joints, spinous processes, posterior ligamentous structures, and paraspinal soft tissues outside the fusion zone.

Decompression at the fracture level was performed when canal compromise, neurological deficit, or retropulsed bony fragments required direct neural element decompression. Rod contouring and reduction maneuvers were used to restore vertebral body height, segmental alignment, and sagittal profile. Excessive distraction across the fused segment was avoided to reduce abnormal loading of adjacent discs and facets [19,20,21]. Posterolateral fusion was performed using bone graft material according to the surgeon’s standard technique. Interbody fusion was added selectively in patients requiring anterior column support or additional segmental stability. Postoperatively, patients were mobilized as early as clinically feasible and were supported with a thoracolumbosacral orthosis for approximately 3 months, unless contraindicated.

Preservation of the adjacent facet joints, posterior ligamentous structures, spinous processes, and paraspinal soft tissues outside the fusion zone represented a shared operative principle among the treating spine surgeons. However, this was not a rigid prospective surgical protocol. The exact extent of exposure, construct length, decompression, reduction maneuver, and need for interbody support were individualized according to fracture morphology, mechanical instability, neurological status, bone quality, and surgeon judgement.

### 2.5. Radiographic Assessment

All patients underwent supine anteroposterior and lateral thoracolumbar/lumbar radiographs, computed tomography (CT), and magnetic resonance imaging (MRI) before surgery when clinically available. Postoperative and final follow-up radiographs were reviewed to assess construct integrity, fusion status, sagittal alignment, junctional changes, and adjacent segment degeneration.

The adjacent segments immediately proximal and distal to the instrumented construct were assessed separately. Radiographic ASD was defined as new or progressive degeneration at either adjacent motion segment compared with baseline imaging. The diagnosis was based on the presence of one or more of the following findings: more than 20% loss of disc height, reduction in intervertebral disc height greater than 3 mm, anterior or posterior listhesis greater than 3 mm, angular instability greater than 5° on dynamic lateral radiographs, new or progressive adjacent disc degeneration, adjacent segment kyphotic change, PJK, DJK, or adjacent-level stenotic/degenerative change consistent with ASD [1,3,8,22,23]. Upper and lower adjacent segment involvement were coded independently, and patients with at least one positive adjacent-level criterion were classified as having radiographic ASD.

PJK and DJK were recorded as junctional phenotypes of adjacent segment failure. PJK was defined as a pathological kyphotic change immediately proximal to the upper instrumented vertebra, whereas DJK was defined as a pathological kyphotic change immediately distal to the lower instrumented vertebra. When serial radiographs were available, progression over time was prioritized over isolated final measurements.

Because radiographic ASD represents a heterogeneous construct, the broad composite endpoint was further stratified in a post hoc hierarchical analysis to improve interpretability. “Any radiographic ASD” was retained as a sensitive composite endpoint for comparison with prior ASD literature. In addition, radiographic findings were categorized as soft degenerative endpoints or hard mechanical endpoints. Soft degenerative radiographic ASD was defined as adjacent disc degeneration or disc-space narrowing without adjacent translation/listhesis, angular change/instability, PJK, DJK, or revision surgery. Hard mechanical radiographic ASD was defined as the presence of adjacent translation/listhesis, angular change/instability, PJK, or DJK. Revision-confirmed clinical ASD was considered the highest severity tier, regardless of the underlying radiographic phenotype.

### 2.6. Definition of Clinical ASD

Clinical ASD was defined as revision-confirmed symptomatic adjacent segment pathology. This definition was intentionally strict and was selected to maximize specificity in a retrospective trauma cohort, in which residual fracture-related pain, implant-related discomfort, neurological recovery, and generalized postoperative disability may overlap with adjacent-level symptoms. Therefore, the term “clinical ASD” in this study refers specifically to surgically confirmed adjacent-segment disease requiring revision surgery. Symptomatic adjacent-segment pathology treated nonoperatively with medication, physical therapy, injections, bracing, or observation was not classified as clinical ASD unless revision surgery was performed. Accordingly, the reported clinical ASD rate should be interpreted as the incidence of revision-confirmed clinical ASD rather than the total incidence of symptomatic ASD.

### 2.7. Assessment of Fusion

Fusion status was assessed using serial radiographs and CT scans when available. Fusion was defined by the presence of bridging trabecular bone across the intended posterolateral fusion mass, absence of progressive radiolucency around the graft or implants, absence of implant failure, and lack of pathological motion at the fused segment on dynamic radiographs when obtained [24,25]. Suspected nonunion was evaluated using CT, particularly in patients with persistent pain, implant loosening, rod or screw failure, or progressive deformity.

### 2.8. Clinical and Patient-Reported Outcome Assessment

Pain intensity was assessed using the visual analogue scale (VAS), recorded at admission before surgery and at the final follow-up. Back-related functional disability was assessed using the ODI. Because all patients had acute traumatic fractures requiring urgent surgical management, the 1-month postoperative ODI was used as the early post-treatment functional reference point rather than as a preoperative score. Final ODI was obtained at the latest available follow-up.

The 1-month postoperative ODI was used as a pragmatic early post-treatment reference point rather than as a true pre-injury or stable functional baseline. Therefore, ODI improvement in this study should be interpreted as recovery from the 1-month postoperative state to final follow-up, not as total recovery from the original traumatic injury or from the immediate preoperative condition.

ODI improvement was calculated as:

ODI improvement = 1-month postoperative ODI − final follow-up ODI.

Thus, positive values indicated functional improvement, whereas negative values indicated deterioration. Final ODI was also categorized using established disability thresholds [26]: 0–20, minimal disability; 21–40, moderate disability; 41–60, severe disability; 61–80, crippled; and 81–100, bed-bound or extreme disability. Achievement of minimal disability was defined as final ODI ≤ 20. Clinically meaningful improvement was evaluated using a primary minimum clinically important difference (MCID) threshold of ≥12.8 ODI points, with sensitivity analyses using ≥10 and ≥15 points [26,27,28].

The main clinical outcome was functional recovery from the 1-month postoperative reference point to final follow-up. Secondary clinical outcomes included final ODI, achievement of ODI MCID, final ODI ≤ 20, final ODI > 40, final VAS, VAS improvement, complications, and revision surgery for clinical ASD.

### 2.9. Observer Reliability

Radiographic assessments were performed independently by three spine surgeons who were not involved in the index surgical care and were blinded to patient-reported clinical outcomes. Disagreements were resolved by consensus before final analysis. Interobserver agreement for categorical radiographic variables was assessed using Fleiss kappa for three-rater agreement and Cohen’s kappa for pairwise comparisons. Reliability for continuous or ordinal-coded radiographic measurements was assessed using two-way random-effects absolute-agreement intraclass correlation coefficients [ICC(2,1)]. Bootstrap 95% confidence intervals were calculated where applicable.

### 2.10. Outcome Measures

The primary radiographic outcome was the occurrence of radiographic ASD at the proximal or distal adjacent segment after posterior instrumented fusion. The primary clinical outcome was ODI-based functional recovery, defined as improvement from the 1-month postoperative ODI to final follow-up ODI. Key secondary outcomes were revision-confirmed clinical ASD, final ODI category, achievement of ODI MCID, final VAS, VAS improvement, perioperative complications, and need for revision surgery.

To explore the clinical relevance of radiographic ASD, patients were compared according to the presence or absence of radiographic ASD. Additional analyses compared patients with and without revision-confirmed clinical ASD. The relationship between ASD and postoperative functional status was assessed using both continuous ODI values and clinically interpretable thresholds, including final ODI ≤ 20 and final ODI > 40.

### 2.11. Statistical Analysis

Statistical analyses were performed using IBM SPSS Statistics for Windows, version 26.0 (IBM Corp., Armonk, NY, USA). Continuous variables were assessed for normality using histograms, Q–Q plots, and the Shapiro–Wilk test. Normally distributed variables were reported as mean ± standard deviation, whereas non-normally distributed variables were reported as median and interquartile range. Categorical variables were reported as frequencies and percentages.

Between-group comparisons were performed according to ASD status. Continuous variables were compared using the independent-samples *t*-test or Mann–Whitney U test, as appropriate. Categorical variables were compared using the chi-square test or Fisher’s exact test when expected cell counts were small. Paired changes in VAS and ODI over time were analyzed using the paired-samples *t*-test or Wilcoxon signed-rank test according to distribution.

The incidence of radiographic ASD and revision-confirmed clinical ASD was calculated as the number of affected patients divided by the total number of eligible patients. ODI improvement was analyzed both as a continuous variable and as a binary clinically meaningful outcome according to MCID achievement. Because revision-confirmed clinical ASD occurred in only 11 patients, conventional multivariable logistic regression for clinical ASD was not performed, as such a model would be vulnerable to overfitting, sparse-data bias, and unstable estimates. Therefore, clinical ASD was analyzed using descriptive statistics, exact/univariable group comparisons, ODI recovery, MCID achievement, and follow-up-duration sensitivity analyses.

Radiographic ASD status was compared using appropriate univariable tests. Associations between ASD phenotype and functional recovery were interpreted as exploratory and hypothesis-generating rather than as independent predictive effects. Sensitivity analyses were performed in patients with at least 24 months and at least 36 months of follow-up and included both incidence estimates and repeated functional outcome comparisons. A two-sided *p* value < 0.05 was considered statistically significant.

## 3. Results

### 3.1. Cohort Characteristics

Ninety-eight patients who underwent posterior instrumented fusion for acute traumatic thoracolumbar/lumbar vertebral fractures were included. The mean age was 40.8 ± 16.6 years, and 69 (70.4%) patients were male. The median follow-up duration was 49.0 months (IQR, 31.5–72.0), with a range of 3 to 251 months. Most injuries resulted from falls from height [60 (61.2%)], followed by traffic accidents [28 (28.6%)]. Surgery was performed urgently, with a median time from admission to surgery of 0.0 (0.0–2.0) days. The median construct length was 5 (4–5) instrumented vertebrae, and interbody fusion was performed in 9 (9.2%) patients. No proximal facet violation was documented. Perioperative complications occurred in 6 (6.1%) patients, all of which were infection related. Baseline and operative characteristics according to radiographic ASD status are shown in Table 1.

### 3.2. Incidence and Radiographic Phenotype of Adjacent Segment Disease

**Figure 1:**
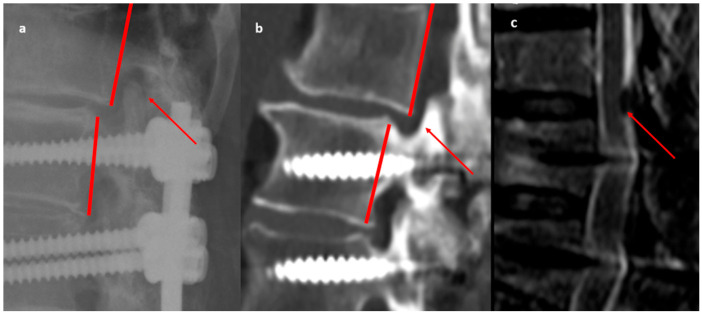
Proximal radiographic adjacent segment disease after posterior instrumented fusion in a 56-year-old man. (**a**) Lateral radiograph demonstrating proximal adjacent-segment listhesis immediately above the upper instrumented vertebra. The red lines and arrow highlight the translational deformity at the supra-adjacent motion segment. (**b**) Sagittal computed tomography image confirming the same proximal adjacent-level malalignment and degenerative structural change above the instrumentation. (**c**) Sagittal magnetic resonance image showing canal compromise and stenosis at the corresponding proximal adjacent level. This case illustrates a proximal radiographic ASD phenotype in which vertebral translation and stenotic remodelling are already visible across complementary imaging modalities, even before the endpoint is necessarily defined by revision surgery.

**Figure 2:**
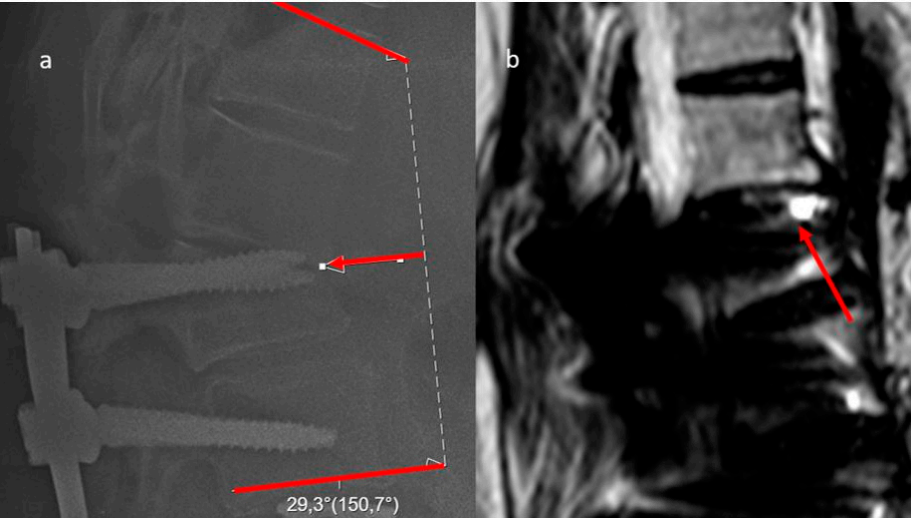
Proximal clinical adjacent segment disease with junctional failure in a 66-year-old woman. (**a**) Lateral radiograph demonstrating proximal junctional kyphosis above the instrumented construct, accompanied by failure of the cranial endplate of the upper instrumented vertebra. The angular marker and arrows indicate the kyphotic transition zone and structural failure at the proximal junction. (**b**) Sagittal magnetic resonance image showing adjacent disc disruption at the proximal junctional level. This figure demonstrates the transition from proximal radiographic junctional change to a clinically relevant ASD phenotype, where kyphotic collapse, endplate failure, and adjacent disc disruption together represent mechanical decompensation of the transition zone.

**Figure 3:**
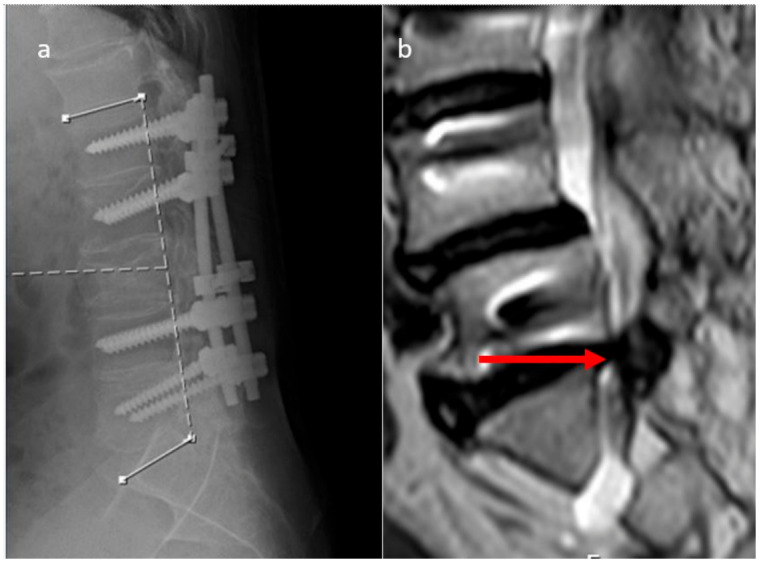
Distal adjacent segment disease associated with postoperative sagittal profile loss in a 60-year-old woman. (**a**) Lateral radiograph showing flattening of the lumbar sagittal profile after posterior instrumentation, consistent with a postoperative flatback-type alignment pattern. This configuration may increase compensatory mechanical demand at the remaining caudal mobile segment. (**b**) Sagittal magnetic resonance image demonstrating distal adjacent-level disc disease and stenosis below the instrumented construct. This case highlights that ASD surveillance after thoracolumbar/lumbar fusion should not be limited to the proximal junction; distal mobile segments may also become mechanically vulnerable, particularly when lumbar lordosis is reduced, and caudal motion segments are required to compensate. The dotted line indicates the reference alignment line used for radiographic assessment, the white line indicates loss of lumbar lordosis as generated by the PACS measurement tool, and the red arrow indicates the relevant distal adjacent-segment pathology.

**Figure 4:**
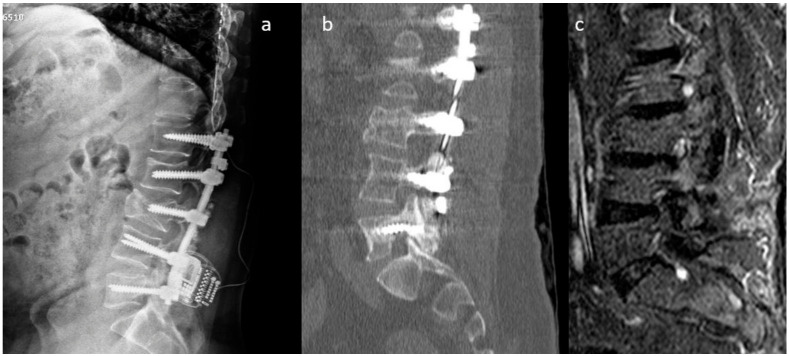
Distal clinical adjacent segment disease with implant-end compromise and severe caudal stenosis in a 58-year-old man. (**a**) Lateral radiograph obtained two years after surgery in a patient with worsening pain, showing long posterior instrumentation and an implanted neuromodulation pulse generator. (**b**) Sagittal computed tomography image demonstrating loosening around the distal instrumentation, widening of the caudal facet joint space, and reduction in disc height at the distal adjacent level. (**c**) Sagittal magnetic resonance image showing severe stenosis at the distal adjacent segment. This case represents a distal clinical ASD phenotype in which pain recurrence, implant-end loosening, facet distraction, disc-height loss, and severe adjacent stenosis converge into a mechanically and clinically meaningful failure pattern.

Radiographic ASD was detected in 54/98 patients (55.1%; 95% CI, 45.2–64.6%). Revision-confirmed clinical ASD occurred in 11/98 patients (11.2%; 95% CI, 6.4–19.0%). All patients who required revision surgery for clinical ASD had radiographic ASD, corresponding to 11/54 (20.4%) of the radiographic ASD subgroup. Proximal/upper adjacent involvement was observed in 39 (39.8%) patients, distal/lower involvement in 36 (36.7%), and combined proximal and distal involvement in 21 (21.4%). The most frequent radiographic phenotype was adjacent disc degeneration or disc-space narrowing [29 (29.6%)], followed by adjacent translation/listhesis [18 (18.4%)], junctional kyphosis [17 (17.3%)], and adjacent angular change [13 (13.3%)] (Figure 1, Figure 2, Figure 3 and Figure 4). The full distribution of ASD phenotypes and hierarchical severity strata is presented in Table 2.

To address the heterogeneity of the broad radiographic ASD composite, a hierarchical endpoint analysis was performed. Forty-four patients (44.9%) had no radiographic ASD. Eleven patients (11.2%) had soft degenerative radiographic ASD only, defined as disc degeneration or disc-space narrowing without mechanical instability, junctional kyphosis, or revision surgery. Thirty-two patients (32.7%) had hard mechanical radiographic ASD without revision, defined by adjacent translation/listhesis, angular change/instability, PJK, or DJK. Eleven patients (11.2%) had revision-confirmed clinical ASD. Thus, the overall radiographic ASD rate of 55.1% should be interpreted as a sensitive composite imaging estimate, whereas mechanically relevant radiographic ASD and revision-confirmed clinical ASD represent more specific severity strata.

### 3.3. Factors Associated with Radiographic ASD

Patients with and without radiographic ASD had comparable age, BMI, follow-up duration, hospitalization duration, construct length, operation time, and estimated blood loss (Table 1). Traffic accidents were more frequent in the ASD group than in the non-ASD group [20 (37.0%) vs. 8 (18.2%); *p* = 0.040]. Perioperative complications were observed only among patients who later demonstrated radiographic ASD [6 (11.1%) vs. 0 (0.0%); *p* = 0.031]. All recorded perioperative complications were infection-related, and only 1 of the 6 patients with a perioperative complication developed revision-confirmed clinical ASD. Diabetes mellitus showed a nonsignificant trend toward higher frequency in the ASD group [18 (33.3%) vs. 8 (18.2%); *p* = 0.091].

### 3.4. Patient-Reported Outcomes and Functional Recovery

Pain and disability improved significantly during follow-up. VAS decreased from 5.4 ± 1.0 preoperatively to 2.7 ± 1.7 at final follow-up, with a mean improvement of 2.7 points (95% CI, 2.2–3.2; *p* < 0.001). ODI decreased from 42.5 ± 15.4 at the 1-month postoperative functional baseline to 27.0 ± 10.9 at final follow-up, with a mean improvement of 15.5 points (95% CI, 12.2–18.8; *p* < 0.001). ODI improvement of at least 10, 12.8, and 15 points was achieved in 49 (50.0%), 39 (39.8%), and 37 (37.8%) patients, respectively. At final follow-up, 4 (4.1%) patients had minimal disability (ODI ≤ 20), whereas 18 (18.4%) had severe residual disability (ODI > 40). Radiographic ASD alone was not associated with a statistically significant difference in final ODI, VAS improvement, or ODI improvement (Table 3).

### 3.5. Revision-Confirmed Clinical ASD and Reduced ODI-Based Recovery

The clinically relevant signal emerged when revision-confirmed clinical ASD was separated from radiographic ASD. Patients with revision-confirmed clinical ASD had markedly smaller ODI recovery than those without revision-confirmed clinical ASD (4.4 ± 2.8 vs. 16.9 ± 17.0; *p* = 0.007). None of the patients with revision-confirmed clinical ASD achieved the primary ODI MCID threshold of 12.8 points, compared with 39 (44.8%) of patients without revision-confirmed clinical ASD (*p* = 0.003). Revision-confirmed clinical ASD was strongly associated with proximal ASD, distal ASD, adjacent translation/listhesis, junctional kyphosis, and distal junctional kyphosis (Table 4). These findings suggest that radiographic degeneration after traumatic fusion may remain clinically silent in many patients, whereas revision-confirmed clinical ASD was associated with attenuated functional recovery from the early post-treatment reference point. This association should be interpreted cautiously and should not be considered evidence that clinical ASD independently caused poor recovery or represents a distinct clinical phenotype.

### 3.6. Sensitivity Analysis According to Follow-Up Duration

Sensitivity analyses were expanded to include both incidence estimates and functional outcomes. Among patients with at least 24 months of follow-up (*n* = 85), radiographic ASD was observed in 44 patients (51.8%) and revision-confirmed clinical ASD in 9 patients (10.6%). The mean ODI improvement in this subgroup was 14.9 ± 16.5 points. Radiographic ASD was not associated with worse final ODI or ODI improvement. ODI improvement was 16.9 ± 17.2 points in patients with radiographic ASD and 12.7 ± 15.7 points in patients without radiographic ASD. In contrast, patients with revision-confirmed clinical ASD showed markedly reduced ODI improvement compared with those without clinical ASD (4.5 ± 3.1 vs. 16.1 ± 17.0), and none of these patients achieved the primary ODI MCID threshold of 12.8 points. Among patients with at least 36 months of follow-up (*n* = 72), radiographic ASD was observed in 36 patients (50.0%) and revision-confirmed clinical ASD in 8 patients (11.1%). The mean ODI improvement was 13.8 ± 16.4 points. Radiographic ASD again showed no consistent association with worse functional outcome. ODI improvement was 16.0 ± 17.2 points in patients with radiographic ASD and 11.6 ± 15.5 points in those without radiographic ASD. In contrast, revision-confirmed clinical ASD continued to identify a subgroup with reduced ODI recovery compared with patients without clinical ASD (4.3 ± 3.3 vs. 14.9 ± 17.0), and no patient with revision-confirmed clinical ASD achieved the 12.8-point ODI MCID threshold. These findings indicate that the principal results remained directionally stable in longer-followed patients.

### 3.7. Interobserver Reliability

Interobserver agreement for categorical radiographic ASD variables was substantial to almost perfect. Fleiss kappa values across the three raters ranged from 0.767 to 1.000. Agreement was almost perfect for upper adjacent disc ASD grade (κ = 0.961; 95% CI, 0.900–1.000), lower adjacent disc ASD grade (κ = 0.951; 95% CI, 0.870–1.000), upper adjacent angular change (κ = 0.958; 95% CI, 0.845–1.000), lower adjacent angular change (κ = 0.925; 95% CI, 0.707–1.000), upper adjacent listhesis (κ = 1.000), lower ad-jacent listhesis (κ = 0.904; 95% CI, 0.796–0.979), and DJK (κ = 1.000). Agreement for PJK was substantial (κ = 0.767; 95% CI, 0.601–0.903). ICC values showed good-to-excellent reliability and ranged from 0.769 to 1.000 using a two-way random-effects absolute-agreement model. These findings support the reproducibility of the radiographic ASD assessments used in the present study.

## 4. Discussion

The present study evaluated adjacent segment disease after posterior instrumented thoracolumbar/lumbar fusion for acute traumatic vertebral fractures and provides three clinically important findings. First, radiographic ASD was common, occurring in 55.1% of patients when a sensitive composite imaging definition was used, whereas revision-confirmed clinical ASD was substantially less frequent, occurring in 11.2%. Second, radiographic ASD alone was not associated with worse final ODI, VAS improvement, or ODI recovery, suggesting that many adjacent segment changes after traumatic fusion remain clinically silent. Third, revision-confirmed clinical ASD was associated with markedly reduced ODI recovery and failure to achieve ODI MCID. These findings support a clinically useful distinction between radiographic ASD and revision-confirmed clinical ASD. The key question is not simply whether adjacent-level degeneration is present, but whether radiographic change is accompanied by surgically confirmed clinical deterioration and reduced ODI-based recovery.

The distinction between adjacent segment degeneration and adjacent segment disease has long been emphasized in the degenerative lumbar fusion literature. Hilibrand and Robbins, and Park et al., described radiographic degeneration and symptomatic disease as related but non-identical outcomes after arthrodesis [1,3]. Ghiselli et al., Radcliff et al., and Lee et al. further showed that adjacent segment pathology is influenced by a complex interaction between natural degeneration, fusion-related biomechanics, and patient-specific susceptibility [2,6,8]. Our results are consistent with this conceptual framework but extend it to a less studied population: patients undergoing urgent posterior fusion for acute traumatic vertebral fractures. In this context, a high radiographic ASD rate should not automatically be interpreted as clinical failure. Instead, our data suggest a two-stage model: a broad radiographic field of adjacent segment stress and a smaller, clinically meaningful subgroup characterized by symptomatic deterioration and revision surgery.

The radiographic ASD rate in our cohort was higher than many rates reported after degenerative fusion and also higher than the 36.4% recently reported after short-segment fixation for traumatic lumbar vertebral fracture [29]. Several factors may explain this difference. Our cohort had a longer median follow-up, included thoracolumbar/lumbar fusion constructs with a median of five instrumented vertebrae, and used a broad radiographic definition that captured disc-space narrowing, listhesis/translation, angular change, and junctional kyphosis. This broad definition was intentionally sensitive, but it should not be interpreted as a homogeneous clinical failure endpoint. The hierarchical analysis addressed this concern by separating soft degenerative radiographic ASD, hard mechanical radiographic ASD, and revision-confirmed clinical ASD. Importantly, the same recent trauma-specific study found no significant VAS or ODI difference between ASD and non-ASD patients during short-term follow-up [29]. This partially supports our finding that radiographic ASD alone may be insufficient to explain patient-reported disability.

Disc-space narrowing or adjacent disc degeneration alone may represent a soft imaging finding and may remain clinically silent. In contrast, adjacent translation/listhesis, angular change, PJK, and DJK represent harder mechanical phenotypes that may better reflect adjacent-segment decompensation. In the hierarchy used in the revised analysis, only 11.2% of patients had soft degenerative radiographic ASD alone, 32.7% had hard mechanical radiographic ASD without revision, and 11.2% had revision-confirmed clinical ASD. Therefore, the main clinical interpretation of the study should not rely solely on the broad composite incidence, but rather on the separation between radiographic burden, mechanical severity, and revision-confirmed clinical failure.

The trauma setting may create a biologically and biomechanically distinct context in which adjacent-segment changes develop. In elective degenerative fusion, adjacent segment degeneration is often interpreted as the consequence of altered motion and increased stress after arthrodesis, superimposed on pre-existing age-related degeneration [1,2,3,8]. In acute vertebral fracture, however, the adjacent segment may already be compromised at the time of injury. Endplate disruption, occult disc injury, posterior ligamentous complex damage, facet capsule trauma, paraspinal muscle injury, local kyphotic collapse, and sudden disturbance of sagittal mechanics may increase the vulnerability of the adjacent disc–facet complex before instrumentation is applied [19,20]. Posterior instrumentation may then further alter load transfer and motion at the nearest unfused segments. Therefore, the present findings should not be interpreted as proof that fusion independently caused ASD, but rather as evidence that radiographic and clinical adjacent-segment changes can emerge within a trauma-specific biomechanical and biological environment.

Our pattern of proximal and distal adjacent involvement also deserves attention. Classic degenerative fusion literature has often emphasized proximal adjacent segment degeneration, particularly after lower lumbar or lumbosacral fusion [7,8,30]. In contrast, trauma-specific fixation may expose both proximal and distal segments to abnormal loading, especially when constructs cross the thoracolumbar junction or terminate near mobile lumbar segments. In our cohort, proximal involvement was observed in 39.8%, distal involvement in 36.7%, and combined proximal–distal involvement in 21.4% of patients. This bilateral adjacent vulnerability is clinically important because distal degeneration after thoracolumbar/lumbar fracture fixation may be underestimated if surveillance focuses only on the upper instrumented vertebra. The association of clinical ASD with translation/listhesis, junctional kyphosis, and distal junctional kyphosis further suggests that not all ASD phenotypes carry the same clinical weight. Disc-space narrowing alone may remain silent, whereas translational or junctional failure may indicate mechanical decompensation.

The use of the 1-month postoperative ODI as the functional reference point requires careful interpretation. In acute traumatic vertebral fracture, a conventional preoperative ODI is methodologically problematic because it largely reflects acute fracture pain, immobilization, emergency admission conditions, and trauma-related distress rather than true baseline disability. For this reason, the 1-month postoperative ODI was selected as an early post-treatment reference point. However, this approach also has limitations. Patients may already have experienced different degrees of recovery during the first postoperative month, depending on injury severity, neurological status, pain control, bracing, wound recovery, mobilization, rehabilitation access, and early complications. Consequently, ODI improvement in this study should be interpreted as recovery from the early post-treatment state to final follow-up rather than as total postoperative recovery. This distinction is particularly important when comparing the present results with studies that use true preoperative, pre-injury, or immediate postoperative functional assessments.

The relationship between ASD and HRQoL after fracture fusion remains underdeveloped in the literature. Recent work has shown that thoracolumbar fusion for traumatic fractures can improve HRQoL and pain, particularly during the early postoperative period [31]. However, fusion constructs may also reduce segmental motion, alter flexibility, and generate persistent disability in selected patients. The systematic review by Wang et al. highlighted the ongoing controversy regarding implant removal after thoracolumbar burst fracture fixation: removal may improve motion and pain in some patients but may also cause kyphotic progression, complications, or additional costs, and current evidence does not support routine removal [18]. Our findings add a complementary perspective. Rather than framing the problem as implant retention versus implant removal alone, the present study suggests that the clinically relevant target should be the identification of the patient whose adjacent segment has become a functional bottleneck. In other words, the decision-making question should evolve from “Is the implant present?” to “Is the adjacent segment mechanically and clinically failing?”

The apparent discrepancy between ODI improvement and final ODI in patients with clinical ASD reflects the importance of analyzing recovery trajectory rather than final status alone. Patients with revision-confirmed clinical ASD had lower 1-month postoperative ODI scores than patients without clinical ASD, but their final ODI scores were numerically higher. As a result, the change from the early postoperative functional state to final follow-up was markedly smaller in the revision-confirmed clinical ASD group. Final ODI alone may therefore obscure clinically meaningful differences in recovery because patients did not begin from identical early postoperative disability levels. The MCID analysis reinforces this interpretation: no patient with revision-confirmed clinical ASD achieved the primary ODI MCID threshold, whereas a substantial proportion of patients without clinical ASD did. Thus, revision-confirmed clinical ASD was associated with reduced ODI recovery rather than merely a higher final ODI score.

The near-threshold *p* values for ODI MCID achievement also require careful interpretation. Patients with radiographic ASD had numerically higher rates of ODI improvement ≥12.8 points and ≥15 points than patients without radiographic ASD, although these differences did not reach conventional statistical significance. Therefore, these findings should not be interpreted as evidence that radiographic ASD worsened functional recovery. The pattern may reflect the numerically higher 1-month postoperative ODI in the radiographic ASD group, which provided greater room for improvement, as well as the heterogeneity of the broad radiographic ASD definition. These borderline findings support the central message that radiographic ASD alone is not equivalent to clinical failure and should be interpreted separately from revision-confirmed clinical ASD.

Several findings should be interpreted cautiously. Traffic accident mechanism and perioperative complications were more frequent in patients with radiographic ASD. These associations are hypothesis-generating rather than causal. A traffic accident may represent a higher-energy injury phenotype with greater occult disc, endplate, facet, and posterior ligamentous complex damage. Similarly, perioperative complications may mark more severe injuries, more complex surgery, delayed mobilization, prolonged inflammatory burden, soft-tissue compromise, or a vulnerable biological host rather than directly causing ASD. All recorded perioperative complications were infection-related, and only one of the six patients with a complication developed revision-confirmed clinical ASD. Therefore, these data do not establish a direct causal relationship between infection and ASD, and the small number of events precluded reliable modelling of complications as an independent predictor.

This study has several strengths. First, it focuses on a homogeneous and clinically relevant population: acute traumatic vertebral fractures treated with posterior instrumented thoracolumbar/lumbar fusion. Second, it separates radiographic ASD from revision-confirmed clinical ASD, preventing overinterpretation of asymptomatic imaging changes. Third, it evaluates both proximal and distal adjacent segments and captures multiple phenotypes, including disc degeneration, translation, angular change, PJK, and DJK. Fourth, it integrates patient-reported functional recovery using ODI improvement and MCID thresholds rather than relying only on statistical significance. Finally, the use of a 1-month postoperative ODI as an early post-treatment baseline is a pragmatic and trauma-appropriate solution to a major methodological problem in acute fracture studies.

This study has several limitations. First, the retrospective single-centre design introduces potential selection bias, information bias, and limited generalizability. Because inclusion required adequate postoperative ODI data and radiographic follow-up, the cohort represents a complete-case surgical follow-up cohort rather than an unselected population-level trauma cohort. Patients with persistent symptoms, implant-related concerns, or radiographic progression may have been more likely to remain under surveillance, potentially overestimating radiographic ASD. Conversely, patients who recovered uneventfully may have been less likely to return for long-term imaging, whereas patients with severe symptoms treated elsewhere may have been missed, potentially underestimating revision-confirmed clinical ASD.

Second, the definition of radiographic ASD was intentionally broad and sensitive. It included disc degeneration, disc space narrowing, listhesis/translation, angular instability, PJK, DJK, and adjacent-level degenerative change. This broad composite definition may increase the apparent incidence of radiographic ASD and should not be interpreted as a homogeneous clinical failure endpoint. Disc-space narrowing or degeneration alone may remain clinically silent, whereas translation, angular instability, PJK, and DJK may represent more mechanically relevant phenotypes. Therefore, the reported radiographic ASD rate should be interpreted together with the separated phenotype and hierarchical severity analyses.

Third, clinical ASD was defined strictly as adjacent-segment pathology requiring revision surgery. This definition increases specificity and reduces the risk of misclassifying nonspecific postoperative pain, residual trauma-related disability, or implant-related symptoms as ASD. However, it may underestimate the true burden of symptomatic ASD because patients treated nonoperatively with medication, physical therapy, injections, bracing, or observation were not classified as clinical ASD. Accordingly, the reported clinical ASD rate should be interpreted as revision-confirmed clinical ASD rather than the total incidence of symptomatic adjacent-segment disease.

Fourth, true pre-injury functional status was unavailable. The 1-month postoperative ODI was used as a pragmatic early post-treatment reference point because acute preoperative ODI in traumatic vertebral fracture primarily reflects fracture pain, immobilization, emergency admission conditions, and trauma-related distress. However, this time point does not represent a stable baseline. Some patients may have already recovered substantially before the 1-month assessment, whereas others may still have been limited by pain, bracing, wound healing, neurological recovery, restricted mobilization, or early complications. Therefore, ODI improvement should be interpreted as recovery from the early postoperative state to final follow-up, not as total recovery from the original injury or surgery.

Fifth, global sagittal and spinopelvic alignment parameters were not systematically incorporated into the analysis. This limitation is partly related to the acute trauma setting. Unlike elective degenerative or deformity surgery, many patients with unstable thoracolumbar/lumbar fractures underwent urgent stabilization and could not safely undergo standardized preoperative standing full-spine radiographs because of pain, mechanical instability, neurological concerns, immobilization, or inability to stand. Therefore, reliable pre-operative measurements of sagittal vertical axis, pelvic incidence, lumbar lordosis, PI–LL mismatch, pelvic tilt, sacral slope, thoracic kyphosis, T1 pelvic angle, and T1 slope were not available across the cohort. For this reason, postoperative-only spinopelvic measurements were not included as covariates in the main analysis. Without a comparable preoperative alignment baseline, postoperative values could reflect pre-existing sagittal morphology, fracture-induced deformity, surgical correction, or postoperative compensation. Including such variables without baseline reference could therefore introduce additional interpretive bias. Nevertheless, unmeasured sagittal malalignment remains an important potential confounder.

Finally, MRI and dynamic radiographs were not uniformly available at every follow-up time point, and the number of revision-confirmed clinical ASD events was limited. This restricted the complexity of multivariable modelling and precluded reliable identification of independent predictors of clinical ASD. The associations involving clinical ASD should therefore be interpreted as exploratory and hypothesis-generating. Future prospective multicenter studies should include standardized imaging intervals, full-length standing sagittal alignment measurements, serial ODI and HRQoL assessment, documentation of symptomatic non-operative ASD, and time-to-event analysis for revision-confirmed clinical ASD.

Future research should focus on prospective, multicenter trauma-specific ASD cohorts with standardized imaging and patient-reported outcome schedules. Baseline MRI should be used to characterize traumatic disc, endplate, facet, and posterior ligamentous complex injury at adjacent levels. Serial ODI, VAS, EQ-5D, return-to-work, analgesic use, nonoperative ASD treatments, and AO Spine PROST should be collected to distinguish radiographic degeneration from clinically meaningful recovery failure. Future studies should also prospectively track all eligible trauma patients from index surgery onward, including those who become asymptomatic or are lost to routine clinical follow-up, to reduce attrition bias. Standardized standing full-spine radiographs should be obtained when clinically safe to incorporate sagittal vertical axis, pelvic incidence, lumbar lordosis, PI–LL mismatch, pelvic tilt, sacral slope, thoracic kyphosis, T1 pelvic angle, and related parameters into ASD and functional outcome analyses. Time-to-event analyses should be used to model clinical ASD and revision risk. Ultimately, the goal should not be merely to document ASD after fracture fusion, but to predict which adjacent segments are likely to remain silent and which are at higher risk of clinically meaningful deterioration.

## 5. Conclusions

Radiographic ASD is frequent after posterior instrumented thoracolumbar/lumbar fusion for acute traumatic vertebral fractures, but most radiographic changes do not translate into measurable functional deterioration. Revision-confirmed clinical ASD was less frequent but was associated with markedly reduced ODI improvement and failure to achieve clinically meaningful recovery. These findings are compatible with a trauma-specific adjacent-segment vulnerability model in which baseline injury burden and postoperative biomechanics may interact. However, causality cannot be established from this retrospective observational study, and the present data do not establish a distinct clinical phenotype. Future long-term surveillance may be strengthened by integrating radiographic assessment with ODI-based recovery, MCID achievement, and HRQoL-centred endpoints, rather than relying on radiographic degeneration alone.

## Figures and Tables

**Table 1:** Baseline demographic, trauma, and operative characteristics according to radiographic ASD status.

Variable	Overall (*n* = 98)	Radiographic ASD (*n* = 54)	No Radiographic ASD (*n* = 44)	*p* Value
Age, years	40.8 ± 16.6	43.3 ± 17.3	37.8 ± 15.4	0.075
Male sex	69 (70.4%)	34 (63.0%)	35 (79.5%)	0.074
BMI, kg/m^2^	29.4 ± 6.1	30.1 ± 6.0	28.5 ± 6.1	0.230
Smoking	28 (28.6%)	16 (29.6%)	12 (27.3%)	0.797
Diabetes mellitus	26 (26.5%)	18 (33.3%)	8 (18.2%)	0.091
Hypertension	16 (16.3%)	11 (20.4%)	5 (11.4%)	0.230
Trauma: Fall from height	60 (61.2%)	30 (55.6%)	30 (68.2%)	0.202
Trauma: Traffic accident	28 (28.6%)	20 (37.0%)	8 (18.2%)	0.040
Trauma: Simple fall	9 (9.2%)	3 (5.6%)	6 (13.6%)	0.292
Trauma: Other	1 (1.0%)	1 (1.9%)	0 (0.0%)	1.000
Time to surgery, days	0.0 (0.0–2.0)	0.0 (0.0–1.0)	0.0 (0.0–2.0)	0.866
Hospitalization, days	6.0 (5.0–10.0)	6.0 (4.2–10.0)	6.0 (5.0–8.0)	0.689
Follow-up, months	49.0 (31.5–72.0); range 3–251	49.0 (30.0–83.0)	49.5 (36.8–61.0)	1.000
Construct length, vertebrae	5 (4–5)	5 (4–5)	5 (4–5)	0.311
Interbody fusion	9 (9.2%)	3 (5.6%)	6 (13.6%)	0.292
Operation time, min	210.0 (180.0–240.0)	217.5 (180.0–240.0)	210.0 (180.0–255.0)	0.801
Estimated blood loss, mL	1400 (712–2400)	1325 (688–2300)	1400 (738–2625)	0.624
Any perioperative complication *	6 (6.1%)	6 (11.1%)	0 (0.0%)	0.031

Values are presented as mean ± SD, median (IQR), or *n* (%), as appropriate. BMI data were available for 96 patients. Construct length was derivable in 97 patients. * All recorded perioperative complications were infection related. ASD, adjacent segment disease; BMI, body mass index.

**Table 2:** Radiographic ASD phenotypes and hierarchical severity strata.

ASD Phenotype/Severity Stratum	*n* (%)
Any radiographic ASD	54 (55.1%)
Proximal/upper adjacent segment involvement	39 (39.8%)
Distal/lower adjacent segment involvement	36 (36.7%)
Both proximal and distal involvement	21 (21.4%)
Any adjacent disc degeneration/disc-space narrowing	29 (29.6%)
Upper adjacent disc degeneration	23 (23.5%)
Lower adjacent disc degeneration	17 (17.3%)
Any adjacent translation/listhesis	18 (18.4%)
Upper adjacent translation/listhesis	3 (3.1%)
Lower adjacent translation/listhesis	18 (18.4%)
Any adjacent angular change	13 (13.3%)
Upper adjacent angular change	9 (9.2%)
Lower adjacent angular change	5 (5.1%)
Any junctional kyphosis phenotype	17 (17.3%)
Proximal junctional kyphosis	14 (14.3%)
Distal junctional kyphosis	3 (3.1%)
Revision-confirmed clinical ASD	11 (11.2%)
Hierarchical severity strata	
No radiographic ASD	44 (44.9%)
Soft degenerative radiographic ASD only	11 (11.2%)
Hard mechanical radiographic ASD without revision	32 (32.7%)
Revision-confirmed clinical ASD	11 (11.2%)

Percentages were calculated using the total cohort as the denominator (*n* = 98). Patients could have more than one individual radiographic ASD phenotype. For the hierarchical analysis, patients were assigned to the highest applicable severity tier in the following order: revision-confirmed clinical ASD, hard mechanical radiographic ASD without revision, soft degenerative radiographic ASD only, and no radiographic ASD. Soft degenerative radiographic ASD included adjacent disc degeneration or disc-space narrowing without translation/listhesis, angular change/instability, PJK, DJK, or revision. Hard mechanical radiographic ASD included adjacent translation/listhesis, angular change/instability, PJK, or DJK.

**Table 3:** Patient-reported outcomes according to radiographic ASD status.

Outcome	Overall (*n* = 98)	Radiographic ASD (*n* = 54)	No Radiographic ASD (*n* = 44)	*p* Value
Preoperative VAS	5.4 ± 1.0	5.4 ± 0.9	5.4 ± 1.1	0.867
Final VAS	2.7 ± 1.7	2.8 ± 1.7	2.5 ± 1.8	0.257
VAS improvement	2.7 ± 2.6	2.5 ± 2.5	2.9 ± 2.8	0.699
1-month postoperative ODI	42.5 ± 15.4	44.6 ± 15.4	39.9 ± 15.1	0.079
Final ODI	27.0 ± 10.9	26.5 ± 10.8	27.5 ± 11.0	0.553
ODI improvement	15.5 ± 16.5	18.1 ± 17.1	12.3 ± 15.3	0.264
ODI improvement ≥ 10 points	49 (50.0%)	28 (51.9%)	21 (47.7%)	0.685
ODI improvement ≥ 12.8 points	39 (39.8%)	26 (48.1%)	13 (29.5%)	0.061
ODI improvement ≥ 15 points	37 (37.8%)	25 (46.3%)	12 (27.3%)	0.053
Final ODI ≤ 20	4 (4.1%)	1 (1.9%)	3 (6.8%)	0.323
Final ODI > 40	18 (18.4%)	9 (16.7%)	9 (20.5%)	0.630
Revision-confirmed clinical ASD	11 (11.2%)	11 (20.4%)	0 (0.0%)	<0.001

ODI improvement was calculated as 1-month postoperative ODI minus final ODI; positive values indicate improvement. Borderline MCIDs should be interpreted cautiously because the numerical direction did not indicate worse recovery in the radiographic ASD group. MCID, minimum clinically important difference; ODI, Oswestry Disability Index; VAS, visual analogue scale.

**Table 4:** Revision-confirmed clinical ASD and ODI-based functional recovery.

Variable	Revision-Confirmed Clinical ASD (*n* = 11)	No Revision-Confirmed Clinical ASD (*n* = 87)	*p* Value
Follow-up, months	61.1 ± 37.0	56.1 ± 39.8	0.517
1-month postoperative ODI	35.2 ± 15.2	43.4 ± 15.3	0.041
Final ODI	30.8 ± 14.3	26.5 ± 10.3	0.115
ODI improvement	4.4 ± 2.8	16.9 ± 17.0	0.007
VAS improvement	3.1 ± 2.7	2.7 ± 2.6	0.843
Any radiographic ASD	11 (100.0%)	43 (49.4%)	<0.001
Upper/proximal ASD	9 (81.8%)	30 (34.5%)	0.006
Lower/distal ASD	9 (81.8%)	27 (31.0%)	0.002
Adjacent disc degeneration	6 (54.5%)	23 (26.4%)	0.054
Adjacent translation/listhesis	6 (54.5%)	12 (13.8%)	0.001
Junctional kyphosis phenotype	5 (45.5%)	12 (13.8%)	0.009
PJK	3 (27.3%)	11 (12.6%)	0.190
DJK	2 (18.2%)	1 (1.1%)	0.033
ODI MCID ≥ 12.8 achieved	0 (0.0%)	39 (44.8%)	0.003
ODI MCID ≥ 10 achieved	1 (9.1%)	48 (55.2%)	0.008
Final ODI > 40	3 (27.3%)	15 (17.2%)	0.419

Clinical ASD was defined as revision-confirmed adjacent segment pathology requiring revision surgery. ODI MCID was calculated from the 1-month postoperative functional reference point to final follow-up. ASD, adjacent segment disease; PJK, proximal junctional kyphosis; DJK, distal junctional kyphosis; ODI, Oswestry Disability Index; MCID, minimum clinically important difference. Note: Interobserver reliability results are reported in the manuscript text and are not repeated as a separate table.

## Data Availability

The datasets generated and analyzed during the current study are not publicly available due to institutional privacy and ethical restrictions but are available from the corresponding author upon reasonable request and with appropriate institutional approval.

## Abbreviations

ASD	Adjacent segment disease
BMI	Body mass index
CI	Confidence interval
CT	Computed tomography
DJK	Distal junctional kyphosis
HRQoL	Health-related quality of life
IRB	Institutional review board
MCID	Minimum clinically important difference
MRI	Magnetic resonance imaging
ODI	Oswestry Disability Index
PJK	Proximal junctional kyphosis
SD	Standard deviation
STROBE	Strengthening the Reporting of Observational Studies in Epidemiology
TLICS	Thoracolumbar Injury Classification and Severity Score
TLSO	Thoracolumbosacral orthosis
VAS	Visual analog scale
